# Intervention Using Body Shadow to Evoke Loading Imagery in a Patient with Complex Regional Pain Syndrome in the Foot: A Case Report

**DOI:** 10.3390/brainsci10100718

**Published:** 2020-10-09

**Authors:** Yoshiyuki Hirakawa, Ryota Imai, Hayato Shigetoh, Shu Morioka

**Affiliations:** 1 Department of Rehabilitation, Fukuoka Rehabilitation Hospital, Fukuoka City, Fukuoka 819-8551, Japan; 2 School of Rehabilitation Osaka Kawasaki Rehabilitation University, Kaizuka City, Osaka 597-0104, Japan; imair@kawasakigakuen.ac.jp; 3 Miura Internal Medicine Michiko Pediatrics Clinic, Kagawa 763-0082, Japan; hayato.pt1121@gmail.com; 4 Department of Neurorehabilitation, Graduate School of Health Sciences, Kio University, Nara City, Nara 635-0832, Japan; s.morioka@kio.ac.jp; 5 Neurorehabilitation Research Centre, Kio University, Nara City, Nara 635-0832, Japan

**Keywords:** complex regional pain syndrome, disgust, body shadow, illusion, loading imagery, gait

## Abstract

We present the case of a female patient who developed complex regional pain syndrome (CRPS) after a right-foot injury. The patient had pain from the right knee to the toes and showed severe disgust at the appearance of the affected limb. Consequently, the affected limb was not fully loaded, and the patient had difficulty walking. General interventions, such as mirror therapy, were attempted, but the effect was limited. We hypothesized that this was due to the disgust toward the affected limb, and we implemented a body-shadow intervention that we developed. This reduced the disgust for the affected limb and improved pain, but neither changed the anticipated pain of loading the affected limb nor improved the patient’s walking ability. The reason for this was considered to be that the previous interventions using the body shadow utilized the third-person perspective, denoting that the image of the load sensation on the sole of the foot during walking was insufficient; therefore, we attempted a first-person body-shadow intervention. The results showed improvement in the patient’s walking ability. In CRPS of the foot, it is important to use interventions that evoke images of loading without causing anticipatory pain, pointing to the effectiveness of body-shadow interventions.

## 1. Introduction

In type 1 complex regional pain syndrome (CRPS), pain is prolonged after a traumatic injury, such as a fracture or sprain. In recent years, effective treatments for CRPS have been studied in the context of pharmacotherapy and physical therapy treatments. In light of this research, early diagnosis and treatment are considered important for effective treatment, but there remains a lack of reliable evidence in support of this [1,2]. CRPS presents with a variety of symptoms, including sensory and autonomic deficits that deviate significantly from the normal healing process, motor deficits such as limited range of motion and muscle weakness, and neglect-like syndrome (NLS) [3,4,5,6], which is a disorder of self-awareness [7]. Posttraumatic stress [8], catastrophic thinking [9], and depression and anxiety [10] are psychosocial factors reported to affect CRPS pain. Moreover, Hirakawa et al. [11] reported a case of CRPS in which pain was affected by aversion for the appearance of the affected limb. Osumi et al. [12] and Turner-Cobb et al. [13] reported that such aversion for the affected limb can increase pain sensitivity [14] and exacerbate pain, and McCabe et al. [15] and colleagues [13] suggested that developing negative feelings regarding the appearance of the affected limb is an important symptom of CRPS. It is also known that dislike of one’s own body alters the body image [16,17,18], and this alteration of the body image has been shown to exacerbate pain [19]. Thus, negative emotions, such as disgust, that patients with CRPS experience for their own bodies alter their body image and are believed to be involved in pain. Generally, however, body-image alteration is associated with pain in patients with CRPS due in part to dysfunction of the inferior parietal lobule, which is responsible for body perceptual abilities [20] and multisensory integration, resulting in a discrepancy between perception and movement [21,22]. For this reason, many interventions for patients with CRPS have been used to reduce pain by reshaping the body image and improving the perceptual and motor discrepancy. Among them, mirror therapy (MT) [23] is a widely practiced intervention that has been shown to effectively reduce pain [24]. However, MT is a method of recreating the body image by use of visual illusion. For this reason, in cases where pain is related to negative emotions, such as disgust for the affected limb, symptoms may be temporarily relieved during MT, but when the patient sees the actual affected area, the pain reappears along with disgust, and the symptoms may not show consistent improvement. It has been reported that treatment with body shadow in such cases resulted in continuous improvement of symptoms [11]. 

The case we report here documents a CRPS patient who had a gait disorder due to severe foot pain and body image disorder [25] and had a strong disgust for the affected area. We performed an intervention in this case using a body shadow, similar to that which was carried out in a previous study [11]. This intervention resulted in reduced pain, but it did not improve her walking ability, as she complained that ‘the thought of loading (the foot) hurts.’ Therefore, we developed a new body-shadow intervention for the treatment of this patient, allowing us to imagine a more subjective and active load. As a result, the patient was able to imagine loading the foot without anticipatory pain and was able to actually load the foot, thereby improving her ability to walk. In this article, we report the details and results of this new clinical intervention using body shadow for gait improvement.

## 2. Materials and Methods

### 2.1. Patient

A woman in her 40s had a right foot contusion in a traffic accident in 2012. The pain and dysfunction in the right foot area did not decrease later, and the patient presented with the following symptoms, fulfilling the Budapest criteria for CRPS diagnosis: continuing pain that was disproportionate to the inciting event, intense allodynia on the foot, sudomotor changes, motor dysfunction (tremor), trophic changes (hair loss), edema of the foot, and significant left–right differences in foot coloration and skin temperature. Subsequently, rehabilitation care was provided at several hospitals, but there was no significant improvement; hence, we started outpatient physical therapy at 2/week in 2018 for pain relief and improvement of functional impairment. Pregabalin and tramadol hydrochloride were administered at the hospital.

The patient had severe pain with allodynia from the right knee joint to the toes [26], and she felt that the right knee joint and below did not belong to her. She complained that ‘I don’t know where my legs are going when I close my eyes’ and was found to have body image disorder and NLS. She wore a shoe-bellied foot and knee brace and walked on a single-sided crutch in daily life. From the start of treatment, mild stretching and strengthening exercises of the ankle joint and MT were performed for 35 days with no significant effect on pain or NLS. 

The patient was informed in writing, and her consent was obtained for publication of this report and the accompanying images. Additionally, approval for this study was obtained from the Fukuoka Rehabilitation Hospital Institutional Review Board (approval no.: FRH2018-R-021).

### 2.2. Assessment of Physical Therapy before the Intervention Using Body Shadow

#### 2.2.1. Pain Assessment

The Short Form McGill Pain Questionnaire 2 (SF-MPQ-2) was used to assess pain. As a result, all pain expressions were found to be as high as 168/220. The patient also had allodynia from the right knee joint to the toes, and the particularly painful toes could not be touched.

#### 2.2.2. NLS Assessment

The NLS was assessed using the assessment table by Frettlöh al [27]. The NLS has two sub-items: cognitive neglect (CN) and motor neglect (MN), where CN is the feeling that the limb does not belong to one’s body, and MN is a symptom that requires special attention and effort to move one’s limbs. The assessment was made by drawing a 100-mm line on a paper, with the left end of the line marked “0,” for “does not apply at all,” and the right end marked “100”, for “applies most strongly”. This method of assessment involves marking the position that the patient indicates as reflecting their symptom severity and measuring the distance from the left side in mm. The instrument includes five items, with higher values indicating more severe NLS symptoms. The present patient had a markedly high NLS total of 475/500 (MN 285/300, CN 190/200).

#### 2.2.3. Disgust Assessment

Negative emotions such as disgust for one’s own limb were assessed using a numeric rating scale (NRS), where “I don’t feel this way at all” was marked with 0 points and “I feel this way very strongly” was marked with 10 points. The patient had an aversion to the affected limb because it was “thinner and worse in color than the better foot (healthy side)” and complained that the intensity of the associated negative emotion was 10 on the NRS.

#### 2.2.4. Body Image Assessment

**Figure 1:**
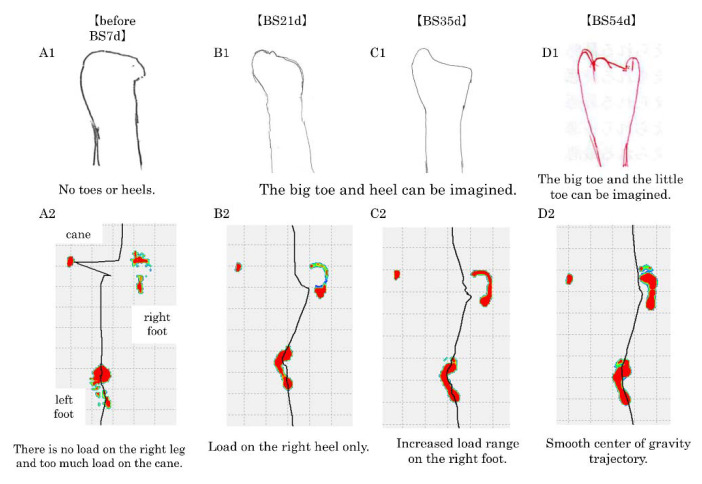
Changes in plantar pressure and center of gravity trajectories during self-drawing and walking. Upper figure; changes in body image (self-portrait of right foot). Lower figure; changes in plantar pressure and center of gravity trajectory. (Upwards is the direction of travel. The black line is the load line. Blue, green, yellow, and red, in this order, indicate stronger pressure.) (**A1,2**): Before body shadow intervention 7 days (before BS2d), (**B1,2**): After body shadow intervention 21 days (BS21d), (**C1,2**): After body shadow intervention 35 days (BS35d), (**D1,2**): After body shadow intervention 54 days (BS54d).

The drawing method was used to evaluate body image [12]. The body image of the foot is shown in Figure 1(A1). When the patient closed her eyes, she complained, ‘My feet are only up to my ankles and I have no toes or heels.’

#### 2.2.5. Gait Performance Assessment

We used the WALK WAY MW-1000 gait analysis system (ANIMA Corporation, Tokyo, Japan) to evaluate gait performance. The WALK WAY is a 9.6 m × 0.6 m sheet-type foot pressure-ground measuring device used to evaluate foot pressure distribution and the trajectory of the center of gravity during walking. The results of the evaluation revealed that the foot pressure distribution during walking showed less loading than usual on the right foot, especially on the toes. Therefore, the cane was overloaded during standing on the right side of the foot (Figure 1(A2)).

### 2.3. Body-Shadow Intervention and Process

First step: interventions aimed at correcting the disgust felt for the affected limb and creating a body image.

**Figure 2:**
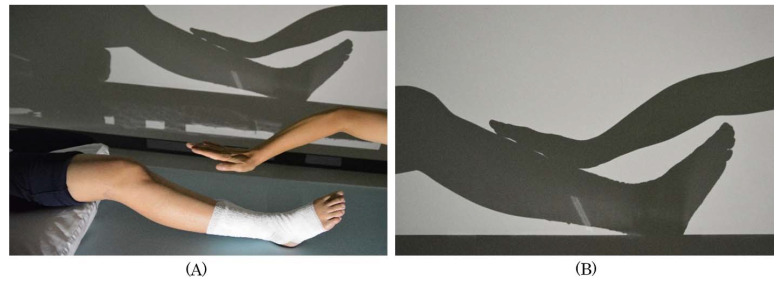
Intervention method using body shadows (first step). A body shadow is created on the wall below the patient’s lower leg in which the physical therapist (PT) appears to be touching the patient’s lower leg. (**A**). The therapist touches the lower legs and feet through the body shadow. (**B**). In this situation, the patient does not feel any pain or fear and can be touched by others.

We created a body shadow by projecting the image of the knee joint to the toe on the wall. When observing the affected limb projected as a body shadow, the patient said: ‘It feels like my own foot, but the color and shape are more abstract than what I actually see, and the size and shape are the same as the size and shape of the better foot (healthy side), so I don’t feel disgusted with it.’ After confirming that the patient did not feel any aversion towards the body shadow, the therapist did not touch the patient, but only touched the patient’s shadow (Figure 2A). In this setting, the therapist appears to be touching the patient’s body, but is only touching the patient’s body shadow (Figure 2). The patient stated: ‘I know my legs because I feel really touched; I know my legs. But I don’t feel any pain, so I’m not afraid.’ This statement confirmed that the sense of body ownership and body image of the right leg, which had been lost, was reconstructed with the help of the illusion of being touched, and no pain or fear was generated. Because of the reconstructed sense of body ownership and body image of the affected lower extremity, the fear of being touched by others on the lower leg and foot was reduced, and the allodynia that was extensively present from the right knee joint to the toes decreased. However, there was no improvement in the patient’s anticipation of pain or fear of grounding the foot, and the patient’s right foot was not fully loaded during walking.

Second step: Interventions aimed at increasing the load on the affected limb.

**Figure 3:**
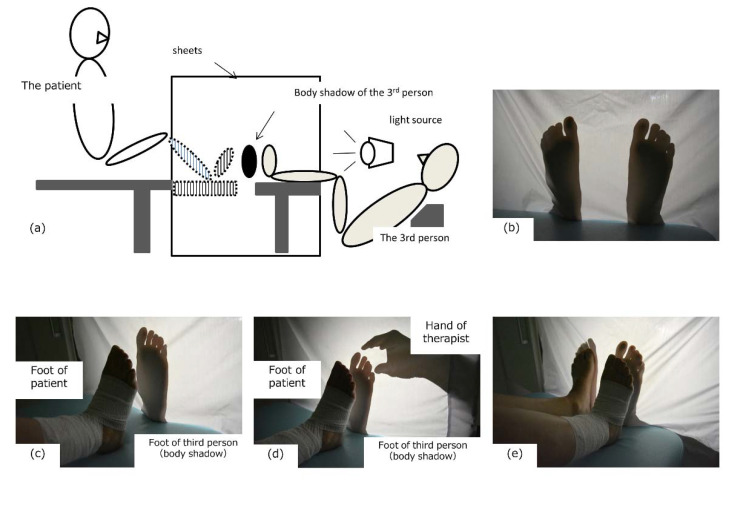
Intervention of the body shadow (second step). When a third person is seated and a sheet is attached to the front of the patient and light is shone from the third person’s side (**a**), the body shadow of the third person’s foot is projected onto the sheet (**b**) (photographed from the patient’s side). When the patient’s foot was observed with the third person’s body shadow, an illusion of the third person’s body shadow and the patient’s own body shadow arose (**c**), and when the therapist touched the third person’s toes, an illusion of being touched by the patient’s own feet arose (**d**). At this time, the patient did not feel any pain and was able to touch her foot to the body shadow by herself (**e**).

The patient complained: ‘I could feel my foot with the body shadow, but I could not imagine stepping on a wall or the ground with my foot. I’m afraid of forcing myself to imagine it and it causes pain in my feet.’ This statement suggested that the reason why the anticipation of pain and fear of plantar grounding did not improve was likely due to inadequate imagery and the anticipatory pain induced by applying pressure to the sole of the foot as if the foot were in active contact with a wall surface and loading. Because the shadowgraph interventions we practiced in the first step were based on the third-person perspective using the body shadow on the wall, we considered it necessary to implement an intervention that would create a stronger image of the body being loaded and would not induce pain or fear. For this reason, we designed a new intervention using the body shadow from a first-person perspective as a second step from the 35th day of the start of body-shadow intervention. This new intervention first involved having the therapist and a third person other than the patient sit face-to-face with the patient. A sheet was placed between the third person and the patient, and light was shone from the third person’s side to create a body shadow of the entire plantar surface of the third person’s foot from the heel to the toe on the sheet (Figure 3a,b). The patient placed her own feet in line with the body shadow of the sole of the third person and observed the body shadow of the third person (Figure 3c). At that time, the patient said, ‘The body shadow of the third person looks like the body shadow of my foot.’ After confirming that the patient felt the illusion, the therapist touched the toes of the third person (Figure 3d). At this point, the patient said, ‘It’s like my toes are being touched, but it doesn’t hurt, strangely enough.’ Next, we asked the patient to touch the body shadow of the third person with her foot and to press her foot with the sole (Figure 3e). Then, she said, ’I can see that I am touching it with the sole of my foot and applying pressure. I could visualize the shape of my toes, and I could imagine putting my weight on the ground, but I don’t feel any pain.’ Hearing this statement, we were able to confirm that the image of the patient loading the sole of the foot without inducing any pain was recalled.

### 2.4. Self-Exercise Instruction

The body shadow illusion enables patients to reconstruct their own body image without pain or fear. Furthermore, because the illusion of body shadow can be easily implemented at home, we taught the patient how to perform home exercises using the body shadow. 

## 3. Results

Table 1 shows the changes over time on the SF-MPQ2, NLS, and disgust for the patient’s own limb, and it also shows the changes over time of the patient’s body image and the distribution of plantar pressure during walking.

From 7 to 21 days after the start of the first step of the body-shadow intervention, SF-MPQ-2 decreased from 168 to 130, 122, and 113, NLS decreased from 475 to 390, 285, and 270, and disgust for one’s own limb decreased from 10 to 7, 5, and 4, respectively. In the NLS, the NLS-CN was high at 190 before the start of the body-shadow intervention, but it markedly decreased to 120 at 7 days after the start of the body-shadow intervention and to 100 at 14 days. Before the start of the body-shadow intervention, it was difficult for the patient to draw the big toe and the heel of the foot, but on the 21st day after the start of the body-shadow intervention, the big toe and heel could be drawn (Figure 1(B1)). Accordingly, the load during walking increased to the heel, and the center of gravity shifted from the cane to the foot (Figure 1(B2)).

From the second step to 54 days after the start of the body-shadow intervention, the SF-MPQ-2 values were 109, 102, and 94, the NLS values were 210, 155, and 145, and the disgust for one’s own limb decreased by 3, 2, and 3, respectively. As for the self-drawing, 35 days after the start of the body-shadow intervention with the addition of the second step, the little toe side could be drawn (Figure 1(C1)), and accordingly, load during walking became possible for the right forefoot (Figure 1(C2)). Fifty-four days after the start of the body-shadow intervention, the big toe and the little toe could be drawn (Figure 1(D1)). During walking, the load on the whole right plantar increased. This allowed for a smoother shift of the center of gravity during walking (Figure 1(D2)).

## 4. Discussion

In the present case, the patient had developed CRPS a long time after a traffic accident and presented with altered body image along with NLS. She had residual severe pain extending from the right knee joint to the toes. In this case, MT, which is generally accepted to be effective, was attempted, but the improvement in symptoms was limited. We consider the following patient statement as the reason for this: ‘The pain decreases when I am using MT, but when I stop using MT, my foot looks ugly again and I dislike my foot.’ Thus, the patient had an aversion to the coloration of the feet and the morphology of the right lower leg, which was thinning due to muscle atrophy. In addition, the patient said that she disliked it because it was ugly.

Therefore, we implemented an intervention using body shadow to create a sense of body ownership and body image without the perception of disfigurement. This body-shadow intervention created a body image without evoking disgust and reduced pain. However, the patient still complained: ‘I could visualize my foot, but I couldn’t imagine stepping on the ground with my foot and loading it, and the image caused pain.’ This suggests that the fear and anticipatory pain caused by contacting the ground with the affected area may have contributed to the residual gait disorder. We considered that the first attempted body-shadow intervention was insufficient to evoke the image of loading because it was performed from the third person’s perspective; thus, we prepared and implemented a new intervention using the body shadow from the first person’s perspective. As a result, the patient was able to recall the image of loading without the expectation of pain, and when the fear of pain decreased, the patient’s gait ability improved.

Over the course of the long-term post-injury period, the patient developed a sense of aversion for the discoloration and muscular atrophy of the affected area, reinforcing the patient’s disgust toward her own body and contributing to the formation of a distorted body image, which is considered a cause of further pain. For this reason, the patient complained that ‘my pain decreases when I’m doing this [MT], but when I look at my leg afterwards, I feel disgusted again and go back to the state I was in before [MT],’ and that the intervention with MT alone did not alleviate the feeling of disgust. On the other hand, the body shadow used in this intervention is unique in that it allows for the abstract visualization of the shape of the affected limb because it projects the shape of the object using only black and white shading. Therefore, when the patient observed the body shadow, she stated: ‘I don’t dislike this shadowed foot because it is the same color and shape as the one on the healthy side.’ Although the patient had an aversion to the color tone and morphology of the affected limb, she did not complain of aversion toward the body shadow of her body because the body shadow of the affected limb was projected in black and white shades only, and she could recognize it as the same color tone and morphology as the healthy limb. This led to an improvement in the feeling of disgust with regard to the affected limb. Gustafsson [28] et al. found that in interventions for patients with chronic pain, it is important to ameliorate shame and disgust for one’s own body and to induce thoughts of respect for one’s own body. However, negative emotions, such as disgust for the affected limb, are not recognized as important symptoms in clinical practice [29]. In contrast, our intervention with body shadow may improve the feeling of disgust caused by the disfigurement of the affected limb. When the limb’s body shadow was observed, the patient replied, ‘I feel like my own foot,’ which suggests that the observation of the body shadow alone did not induce a sense of disgust toward the limb and also induced a sense of body ownership.

It has been shown that body shadow induces the illusion of contact with the affected limb on the body shadow and that the body perceptual ability and the sense of possession of the limb are induced by the observation of the body shadow [30]. Furthermore, it has been shown that the illusion of being touched on the body shadow can be achieved without actual tactile input [31,32]. In this case, we believe that observing the body shadow reflected in the black and white shades of the body provided a sense of body ownership that was not accompanied by negative emotions for the limb’s appearance, and, furthermore, it was considered that it was possible to promote the reconstruction of body image by inducing the illusion of contact that did not induce pain. This is a characteristic of the intervention method using body shadow. However, the first step of the intervention did not improve the anticipatory pain and fear, and it was difficult to actively touch the sole of the foot to the ground. It has been reported that patients with CRPS experience pain even in the absence of nociceptive stimuli by observing movements and images that they themselves expect to induce pain [33]. This anticipatory pain is considered to be caused by the same brain activity that occurs when one expects to experience pain and actually experiences it [34,35,36]. For such symptoms, we considered it necessary to form an image of the loading on the sole of the foot in a way that would not cause unexpected pain, and, as a second step, we devised an intervention method using a body shadow from the first-person perspective.

It has previously been reported that the first-person perspective is important for the training of the image of loading and walking [37,38]. The method used in this study enabled us to observe the body shadow of the third person’s sole from the opposite side of the patient’s body, which enabled us to observe the third person’s body shadow from a first-person perspective. This gave the impression that the body shadow’s feet were the patient’s own feet, and, thus, a sense of body ownership was induced. Furthermore, observing the therapist touching the body shadow of a third party, rather than the patient herself, elicited a painless contact illusion to the patient. This illusion of a third party’s body is a method termed the virtual body swapping illusion (VBSI). It is important that the VBSI not only matches visual and somatosensory information, but also provides the first-person perspective to the body, creating the illusion, and that the patient receives tactile stimuli synchronized with the tactile stimuli to the virtual body [39,40]. Our intervention method using body shadow was based on these conditions. This allowed active load imagery to take place without anticipatory pain. Thus, our new body-shadow intervention improved allodynia in the foot by allowing the patient to experience the VBSI and observe the other person’s body shadow from a first-person perspective, thereby leading to a sense of body ownership and body image. This reduced the anticipatory pain and fear of actively loading the affected plantar on the wall or ground, which led to an improvement in walking ability. The negative feelings toward the patient’s own limb in the present study were formed and reinforced over a long period of time. The body shadow method has the advantage that it is easy to perform at home if there is a dark room and a light source. The patient was able to actively practice at home, and it is considered that this led to improvement in her symptoms.

The treatment of patients with CRPS requires a multimodal intervention tailored to the individual patient’s condition. The body-shadow intervention may be an effective intervention for patients who are disgusted with their bodies. On the other hand, there are a number of issues that need to be addressed, such as the criteria for using body-shadow interventions and whether or not there may be side effects that might be contraindicated.

## 5. Conclusions

We encountered and documented here a case of CRPS in which the patient presented with a gait disorder due to anticipatory pain and fear of loading the affected limb. We implemented a new treatment method using a first-person perspective body-shadow intervention to help the patient more actively imagine the simulated sensation of loading. This enabled the patient to imagine loading without pain. Her fear of loading was thereafter reduced, which improved her ability to walk. Based on our findings, we conclude that a body-shadow intervention could be effective for patients with CRPS since it may allow them to visualize the load required for walking without anticipating experiencing any pain.

## Figures and Tables

**Table 1:** Neglect-like symptoms (NLS) and disgust for the affected lower limb.

	Before BS35d	Before BS7d	BS7d	BS14d	BS21d	BS35d	BS49d	BS54d
SF-MPQ-2 (total)	166	168	130	122	113	109	102	94
SF-MPQ-2 (continuous pain)	44	46	40	33	33	26	22	20
SF-MPQ-2 (intermittent pain)	42	41	26	23	22	22	22	20
SF-MPQ-2 (neuropathic pain)	52	52	42	49	40	47	46	44
SF-MPQ-2 (affective descriptors)	28	29	22	17	18	14	12	10
NLS (total)	480	475	390	285	270	210	155	145
NLS-MN	290	285	270	185	175	150	125	120
NLS-CN	190	190	120	100	95	60	30	25
Disgust assessment	9	10	7	5	4	3	2	4
Rehabilitation program								
Static stretch								
Muscle strength training								
Mirror therapy								
Body-shadow intervention, first step								
Body-shadow intervention, second step								

SF-MPQ-2 = Short-Form McGill Pain Questionnaire 2, NLS = neglect-like symptoms, MN = motor neglect, CN = cognitive neglect, BS = body shadow, d = day, Gray bars indicate therapy delivery timeline.
